# Impact of chronic and acute academic stress on lymphocyte subsets and monocyte function

**DOI:** 10.1371/journal.pone.0188108

**Published:** 2017-11-16

**Authors:** Viktoriya Maydych, Maren Claus, Nicole Dychus, Melanie Ebel, Jürgen Damaschke, Stefan Diestel, Oliver T. Wolf, Thomas Kleinsorge, Carsten Watzl

**Affiliations:** 1 Department of Psychology and Neurosciences, Leibniz Research Centre for Working Environment and Human Factors at TU Dortmund, IfADo, Dortmund, Germany; 2 Department of Immunology, Leibniz Research Centre for Working Environment and Human Factors at TU Dortmund, IfADo, Dortmund, Germany; 3 Institute of Cognitive Neuroscience, Ruhr-University Bochum, Bochum, Germany; Queen Mary University of London, UNITED KINGDOM

## Abstract

This study investigated the effects of a temporally confined naturalistic stressor (academic stress) on immune functions. Furthermore, moderating influences of a number of psychological variables were assessed. Five blood samples were obtained from 20 students during an observation period of 8 weeks, starting 4.5 weeks before an exam period up to 1 week following the last exam. The analysis of 45 immune parameters revealed several time-dependent changes attributable to examination stress. We observed a reduction in the absolute numbers of natural killer (NK) cells and monocytes in peripheral blood and a shift towards more immature and naïve cells within NK and T cell populations. In addition, IL-6 and TNF-α production by LPS-stimulated monocytes was increased. Psychological variables were grouped by means of factor analyses into two factors. One factor, which was interpreted as an indication of chronic stress, moderated the relationships between academic stress and percentages of mature CD57^+^ NK cells. This chronic stress factor was also associated with an increase in memory and a decrease in naïve CD8 T cells and increased serum levels of IL-17. The present study identifies important potential psychological mediators of stress-induced changes in specific immunological parameters.

## Introduction

Stress is an integral part of modern life. Stressful situations comprise a wide range of internal or environmental conditions or events, e.g. bereavement, caregiving for a relative with chronic disease, interpersonal conflicts, juggling many roles and responsibilities, job strain, unemployment, financial worries, over-exercising, and many others. Effects of psychological stress on immune functions have been demonstrated in numerous studies (for reviews see [1, 2]). Typical results include a reduction in the number and cytotoxicity of Natural Killer (NK) cells [3, 4], decreased percentages of CD4 helper T cells and CD8 cytotoxic T cells [5, 6], elevations of antibody titers to various herpes viruses [6, 7], as well as lower lymphocyte proliferation in response to specific mitogens [8, 9], all indicating detrimental effects on cellular immunity and immune function more broadly. These effects vary depending on the type of stressor studied. Furthermore, studies differ in the immune parameters that are examined, which make the results rather difficult to compare. In addition, there might be inter-individual differences in psychological responsiveness to stress, which may moderate the effect of stressful life experiences on immune functioning.

Academic stress is one research paradigm used to investigate the effects of psychological stress on the immune system [10]. Facing academic examinations belongs to real-life challenges that induce a certain amount of stress in most individuals. Academic stress, in psychoneuroimmunological studies often referred to as a brief naturalistic stressor, can be conceptualized as a type of stressor having both acute (e.g. immediately before and during an exam) and prolonged characteristics (e.g. during the preparation or review period) [2, 11]. Thus, academic examinations fall somewhere in-between the continuum between acute and chronic stress. Studies, which examine the relationship between examination stress and immune parameters, usually use some repeated measurement design to compare the pre-exam and post-exam immune status of students [7, 9, 12–15]. Most studies compare the baseline immune status measured some weeks prior to examinations with the immune status measured one day before or shortly after examinations. However, most studies do not assess immune parameters during the extended periods of examination stress, namely during the anticipation of stress and the post-exam period when waiting, possibly anxiously, for results.

According to contemporary conceptions of the nature of stress, three major components can be distinguished: the presence of a stressor (1), subjective appraisal of this stressor as harmful or aversive (2), and the stress response (3) [16]. While physical stressors elicit a stress response rather directly, psychological stressors first require a cognitive appraisal by the individual, which then elicits a response. Thus, depending on the subjective perception and interpretation of a stressor due to previous experiences and coping strategies, responses to stress can be different [17]. Based on inter-individual differences in reactivity to stress [18], it is obvious that immune responses to stress also vary between people. Few studies investigated these inter-individual differences in immune functioning specifically with regard to the effects of brief naturalistic stress. For example, the frequency of engaging in relaxation practice was shown to increase the percentages of T helper cells on the day of an examination and result in higher numbers of T and B lymphocytes during the examination period [15]. Poorer NK cell activity during examinations could be predicted by loneliness [19], emotional instability and high anxiety [20]. Psychological characteristics associated with resiliency may protect individuals against immune suppression or dysregulation in response to academic stress [21]. A potential role of further cognitive factors in immunological processes, including cognitive states and beliefs, is a relatively new and under-investigated area in psychoneuroimmunology. Only few studies have systematically examined the role of affective and cognitive factors on immune responses during brief naturalistic stress related to academic examinations. Considering psychological moderators of the stress-immunity relationship could shed light on mechanisms that are not captured by the main effects described in psychoneuroimmunology studies and may therefore help in clarifying some of the hitherto heterogeneous results.

The present study was designed to meet two objectives. First, we aimed at tracing the time course of eventual changes of immune functioning during an examination period. Therefore, we employed a design with five repeated measurements covering not only the period of acute examination stress but also the pre- and post-examination period. We determined several immune parameters, including total leukocyte counts, a phenotypic analysis of NK and T lymphocyte populations and measurements of cytokines.

Second, we were interested in possible modulations of stress-induced changes in immunological parameters by psychological factors. The psychological assessment in our study captured a series of psychometric questionnaires often used with regard to stress in work-related contexts. The traits and behavioral tendencies we measured were earlier shown to be associated with attenuation of negative effects of stress (i.e. active coping, expectation of success, self-control capacity), stress-related psychiatric diseases (i.e. depression), and stress-related states (ego depletion, general affective states) [22, 23]. Some psychological variables, depressive symptoms, burnout, positive and negative affect, active coping, emotional coping, and personal strain, have been previously reported to be related to immune functioning [24, 25]. Psychological scales ego depletion, capacity of self-control, expectation of success, and job demands have been taken into account on the basis of general theoretical considerations. To our knowledge, there is no study that was concerned with linking these psychological concepts from occupational psychology to immune parameters. In a wider context evidence of this kind of associations can help to evaluate and prevent stress-related health risks in the context of work. These analyses were primarily explorative in nature and should serve to guide future research delving deeper into the mechanisms by which psychological factors affect the responsiveness of the immune system to stress.

## Materials and methods

### Participants

Study participants were 39 undergraduate students, recruited at the Ruhr University of Bochum, Germany. Participants were only included if they (a) fluently spoke German, (b) were not taking medication influencing immune functions, (c) were not pregnant, and (d) had no reported neuropsychological or psychiatric illnesses. They received either a total of 150€ or course credits for their participation. Three students dropped out of the study. One participant was excluded because her/his Depressive Symptoms score was 3 standard deviations above the mean, indicating clinical depression. Another participant was excluded because her/his age was 2 standard deviations above the sample mean. As age is known to influence immunity, including this participant would have distorted the homogeneity of our sample.

Furthermore, due to problems in blood sampling at individual times of measurement, complete data sets could only be obtained from 20 participants (85% female), ranging from 19 to 25 years of age (mean age 22.4, SD = 2.09). All subjects gave written informed consent to participate at the study. All procedures of the study were approved by the local ethics committee of the Leibniz Research Centre for Working Environment and Human Factors.

### Procedure

Participants arrived at the laboratory between 9 a.m. and 2 p.m. They first filled out a questionnaire that assessed their general health and health-related behaviors. Participants also were asked whether they drank alcoholic beverages on the day preceding the measurement. In the next step, participants were asked to complete a psychometric test battery (see below). After completing questionnaires blood samples were drawn.

**Fig 1:**
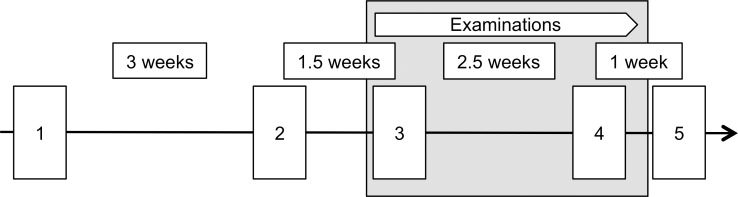
Study design. Study participants were 1^st^ and 2^nd^ year students studying at the Faculty of Psychology (Ruhr-University Bochum, Germany). The first two sessions took place 4.5 weeks and 1.5 weeks before the examination period, at the beginning and at the end of January. The third session was scheduled for the first day of the examination period, in the middle of February. The last two sessions took place directly after and one week after the examination period, at the end of February and at the beginning of March, respectively. Questionnaires were applied to assess general health and health-related behaviors and stress-related psychological parameters. Further, blood and saliva samples were taken.

Apart from the assessment of demographic variables (sex, age, field of study), which took place only once, participants underwent the same procedure five times (Fig 1). The first two sessions took place 4.5 weeks and 1.5 weeks before the examination period, at the beginning and at the end of January. The third session was scheduled for the first day of the examination period, in the middle of February. The last two sessions took place directly after and one week after the examination period, at the end of February and at the beginning of March, respectively.

### Psychological assessment

Without being committed to specific theoretical assumptions, for pragmatic reasons we conceptually distinguished between more stable or trait-like measures on the one hand and more state-like psychological measures on the other hand [25]. We considered depressive symptoms, burnout, self-control capacity, job demands, and private strain as more stable traits, which we expected not to be affected by examination stress. These scales were measured once at session 1 and used as predictors for stress-related changes of immune parameters. On the other hand, the psychological variables ego depletion, general affective states, active and emotional coping, and expectation of success are related to emotional states, and can therefore vary due to various situational factors like, inter alia, examination stress. We assessed these psychological state measures repeatedly. In the following, we report detailed scales’ descriptions and indicate when these were measured.

#### Burnout

The two burnout dimensions Emotional Exhaustion and Depersonalization were assessed by Büssing and Perrar’s (1992) [26], German translation of the Maslach Burnout Inventory [27]. Exhaustion (nine items) addresses feelings of being overextended and drained by job demands. This dimension was adapted to the academic domain (e.g., ‘I feel emotionally drained from my study’). Depersonalization (four items) is characterized by a detached, indifferent, and cynical attitude towards people with whom one has to interact at work. Two items of this dimension were also rephrased by using the word "study" instead of "work" (e.g., ‘I have become more callous towards people since I am studying). All items are scored on a 6-point rating scale (1 = not at all, 6 = very strong). Burnout was measured once at session 1.

#### Depressive symptoms

Depressive symptoms were measured with a shortened, German version of the Beck Depression Inventory [28]. The 15 items refer to various symptoms such as sadness, reduced initiative, hopelessness, irritation, tiredness etc. [29]. Intensity/severity of symptoms is rated by a 6-point frequency rating format (0 = never, 5 = very often). We assessed depressive symptoms once at session 1.

#### General affective states

We measured general affective states using a German translation of the Positive and Negative Affect Schedule (PANAS) of [30]. Participants were asked to rate the intensity of experiencing each out of 20 emotions on a 5-point Likert Skale ranging from 1 (very slightly) to 5 (very much) in the last 12 months. Half of the presented emotion words relate to positive affect (e.g. alert, excited, enthusiastic, inspired, proud), the other half to negative affect (e.g. upset, guilty, ashamed, irritable, scared). General affective states were measured at each session (5 times in total).

#### Active coping

Active coping was assessed by a custom seven items scale phrased based on Latack and Havlovic (1992) [31] and covering strategies aimed at creating favorable preconditions for meeting the demands addressed (e.g. ‘If something bothers me at my study, I try to switch it off as quickly as possible’; ‘Before tackling difficult tasks, I try to keep away from all possible disturbances’). All items are scored in a five-point Likert-rating format (1 = not all, 5 = a great deal). The item scores were averaged to obtain a scale score. The internal consistency of that scale was α = .55. Active coping was assessed at each session (5 times in total).

#### Expectation of success

Expectation of success was measured with a custom scale of 10 items addressing expectations regarding the likelihood of (successfully) passing exams (e.g. ‘All in all I will succeed in upcoming exams’). All items were scored in a 5-point intensity rating format (1 = not at all, 5 = a great deal). The item scores were averaged to obtain a scale score. Cronbach’s alpha for this scale was α = .91. We measured expectation of success at sessions 1, 3 and 4.

#### Emotional coping

Emotional coping was measured with a custom scale of five items referring to “managing the emotions that accompany the perception of stress” (e.g. ‘Even If I am extremely irritated of my study I try to stay calm and relaxed’, ‘I try to take pleasure even in unpleasant tasks in my study.’ All items were scored on a five-point Likert-rating format (1 = not all, 5 = a great deal). The item scores were averaged to obtain a scale score. The internal consistency of that scale was α = .7. Emotional coping was assessed at each session (5 times in total).

#### Self-control capacity

SCC as an individual trait was measured by a German translation of the self-control scale developed by Tangney et al. (2004) [32, 33]. The scale concerns various aspects of self-control, in particular control over thoughts, emotional control, impulse control, performance control, and habit breaking (e.g. ‘People would describe me as impulsive’, ‘I often interrupt people’). Participants were asked to rate items on a five-point rating scale ranging from 1 (not at all) to 5 (very much). The internal consistency for this measure was α = .84. SCC was measured once at session 1.

#### Job demands

Job demands were assessed with items from the job scales developed by Prümper et al. (1995) [34]. 3 items refer to quantitative workload, addressing job demands like ‘time pressure’ and ‘large amount of work’. 3 items refer to qualitative workload, consisting of statements referring to ‘high demands on concentration’ and ‘high variety of tasks’. All items are scored on a 5-point rating format (1 = totally incorrect—5 = totally correct). Participants were asked to indicate the extent to which the respective statement applies to their studies on a five-point Likert-scale ranging from 1 (totally incorrect)–5 (totally correct). The internal consistency for this measure was α = .83. Job demands were measured once at session 1.

#### Private strain

Private strain was measured by a 16 items custom scale based on Kanner et al. (1981) [35], covering strain related to different life domains like family (e.g. ‘lack of time for the family’, ‘troubles with children’), financial troubles (e.g. ‘financial uncertainty’, ‘financial responsibility for others’), personal troubles (e.g. ‘worries about health’, 'worries about inner conflicts’). Respondents were first asked to indicate whether they experienced the respective kind of trouble, and afterwards to indicate the severity to which they experienced it on a four-point-scale ranging from 0 (not at all)– 4 (very severe). Cronbach’s alpha for this scale was α = .65. Private strain was assessed once at session 1.

### Biological analysis

Saliva was collected to assess free cortisol concentrations [36] as a marker of HPA axis activity. The samples were collected using Salivette sampling devices (Sarstedt, Nümbrecht, Germany). Participants collected saliva in the late evening (23:00h) and the next morning of the day following the university appointment. Saliva was collected upon awakening and thirty minutes later in order to assess the cortisol awakening response [37]. Free cortisol concentrations were analyzed without prior extraction using a commercial Chemoluminescence Immunoassay (CLIA; IBL International, Hamburg, Germany). All inter- and intra-assay variations were below 10%. A complete set of cortisol data for all 5 session could be obtained from 29 participants.

Immunological phenotyping was performed using blood samples as recently described [38]. Using the methods described in this publication we determined the absolute number of T cells, B cells, NK cells and monocytes in whole blood. Using flow cytometry we performed an analysis of NK and T lymphocyte subpopulations. We stimulated whole blood samples with Lipopolysaccharide (LPS) and measured the production of IL-6 and TNF-α. Finally, we determined the concentrations of cytokines in serum samples. The biological functions of the different immune parameters investigated in this study are detailed in a recent publication [38].

### Statistical analyses

Initially, we confirmed that age was not correlated with any immunological parameter at any point of measurement. Therefore, age was not included as a control variable. Statistical analyses proceeded in several steps tailored to the study objectives as outlined above. In a first step, we analyzed stress-induced changes separately by repeated measurement analyses of variance (ANOVAs) with five levels of the within-participants variable Session and the respective immune parameter and salivary cortisol level as the dependent variable. We applied Greenhouse-Geisser corrections where appropriate. Significant effects of Session were followed up with Fisher's least significant difference tests (LSD).

In the second step, we analyzed stress-induced changes separately by repeated measurement analyses of variance (ANOVAs) with five levels of the within-participants variable Session and the psychological state variable as the dependent variable. We applied Greenhouse-Geisser corrections where appropriate. Significant effects of Session were followed up with Fisher's least significant difference tests (LSD).

In a third step, we submitted the questionnaire scales of the psychometric measurements to a Factor analyses. This analysis served two functions. First, it facilitated the interpretation of our data. Second, we aimed at dealing with the problem of in part substantial inter-correlations among the psychometric measures as detailed below.

In a fourth step, we analyzed inter-individual differences in stress-induced changes by 2x5 ANOVAs with dichotomized factor scores based on median splits and the five-level within-participants variable representing Session that captured the time course of examination stress. Statistically significant interactions were followed up by tests of simple effects for the assessment of statistical significance between groups on each level of the factor Session and Fisher's least significant difference (LSD) for assessment of statistical significance between levels of the factor Session within each group.

In a fifth step, we computed correlations between psychometric predictors and immunological parameters or salivary cortisol levels at each individual session, as well as correlations between percentage change values of psychometric variables and percentage change values of both immunological parameters and salivary cortisol levels. Since session 1 took place prior to the beginning of the examination period, the relationships to that point of time were considered to imply some kind of basic inter-individual differences in immunity depending on psychological variables. Again, correlations at Sessions 2 to 5 and correlations between percentage change values are assumed to reflect changes to the relationships between immunological parameters, salivary cortisol levels, and psychological parameters under stress.

## Results

To investigate the effect of anticipated and acute stress on the immune system, we studied a group of healthy volunteers consisting of 39 undergraduate students of the Ruhr University Bochum in Germany. These students were undergoing a two-week period where they had to complete several study exams. To investigate changes of the immune system in preparation for, during, and after this stressful exam period we examined the students at five different sessions over a period of 8 weeks (Fig 1). In addition to a psychometric test battery, we took saliva samples to measure cortisol levels and blood samples to determine immunological parameters [38].

Cortisol levels at 30 minutes after awakening showed an increase during the first three sessions, which were before the exam period, and stayed high for the last two sessions after the exam period (F(4, 112) = 1.76 p = .142) (S1 Fig). The cortisol levels at the time point of awakening (F(4, 112) = 3.05 p = .020; post-hoc LSD tests showed a significant increase from Session 2 to Sessions 3 (p = .001) and 5 (p = .026), and a significant decrease from Session 3 to Session 4 (p = .026)) and the cortisol awakening response (CAR) (F(4, 112) = 3.05 p = .020; post-hoc LSD tests demonstrated a significant increase from Session 1 to Sessions 2 (p = .013), 4 (p = .018) and 5 (p = .046)) did both show significant differences over the observation period. To compare our results with previous studies, we conducted additional 2x2 ANOVA with Session (session 1 = no examination stress, session 3 = examination stress) and absolute values of morning cortisol (awakening, awakening + 30 min) as within-subject factors. We found a trend towards a main effect of session (F(1, 28) = 3.2; p = 0.085, r = .319), such that cortisol levels (awakening, awakening + 30 min) were higher at the examination stress session than at the non-stress session. The effect size of r = .32 can be gauged as a medium effect of examination stress on cortisol which is in line with effects reported by others [39–41].

**Fig 2:**
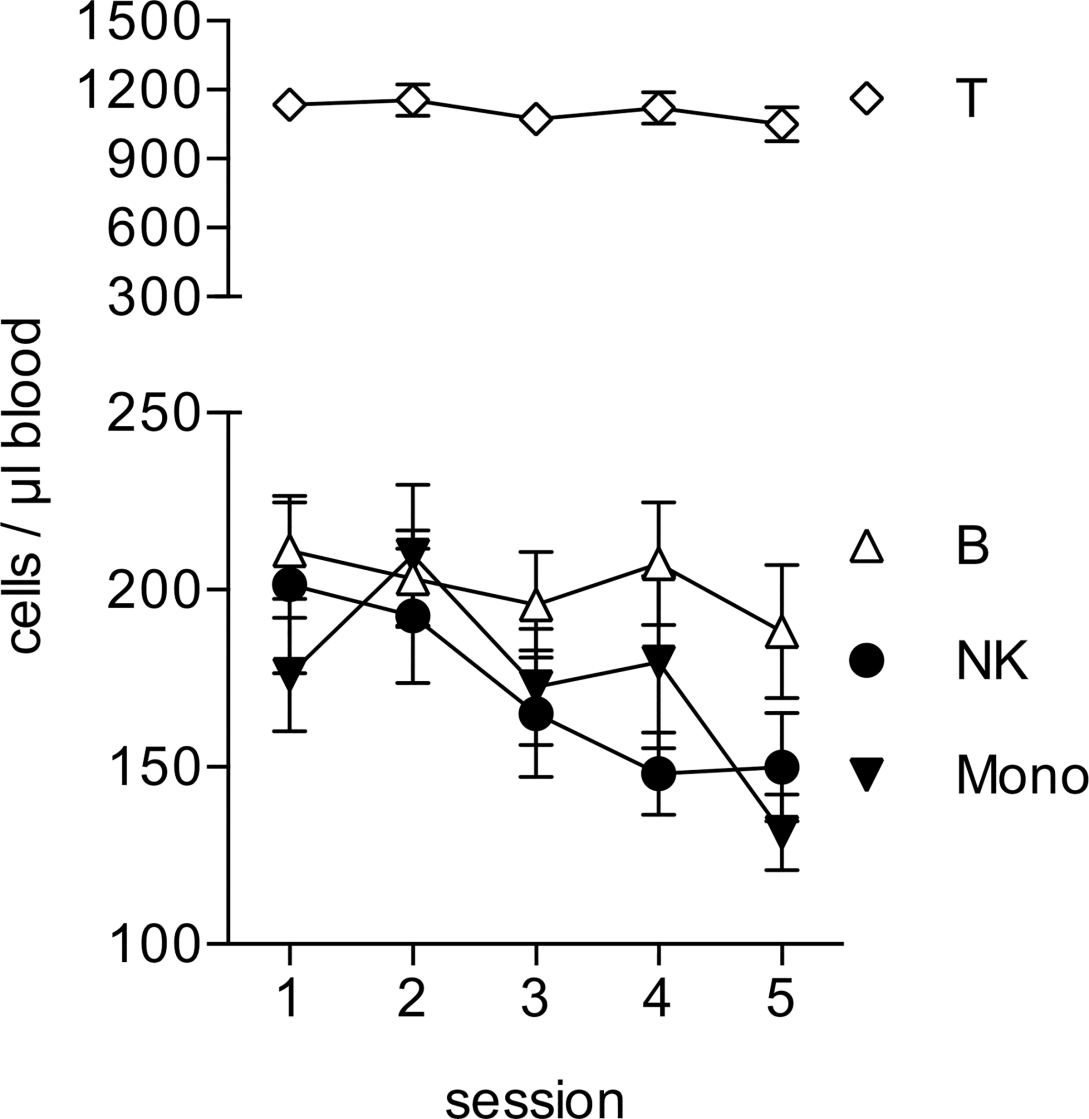
Changes in the absolute numbers of leukocytes. The absolute numbers of T cells, B cells, NK cells and Monocytes were determined in fresh whole blood by flow cytometry using TruCount Tubes. Data from each session are presented as means ± sem of 20 individual participants. Data were analyzed by repeated measures ANOVA. Data sets displaying significant differences between sessions are shown in black.

Analyzing the absolute numbers of lymphocytes and monocytes in the blood samples, we found that the number of Natural Killer (NK) cells showed a significant decrease during the preparation period (sessions 1–3) and stayed low after the exam period (F(2.650, 50.356) = 4.86 p = .006). LSD post-hoc tests demonstrated a significant mean difference between Sessions 2 and 4 (p = .044) (Fig 2). We found a similar change for the number of monocytes, which showed its lowest value at the last session one week after the exam period F(4, 76) = 6.71 (p = .000). LSD post-hoc tests demonstrated a significant mean difference between Sessions 2 and 5 (p = .002). These changes were specific, as we did not find any significant changes in the absolute numbers of T- or B-lymphocytes (F_T_ (2.982, 56.650) = 1.30 p = .282; F_B_ (4, 76) = 1.34 p = .263).

**Fig 3:**
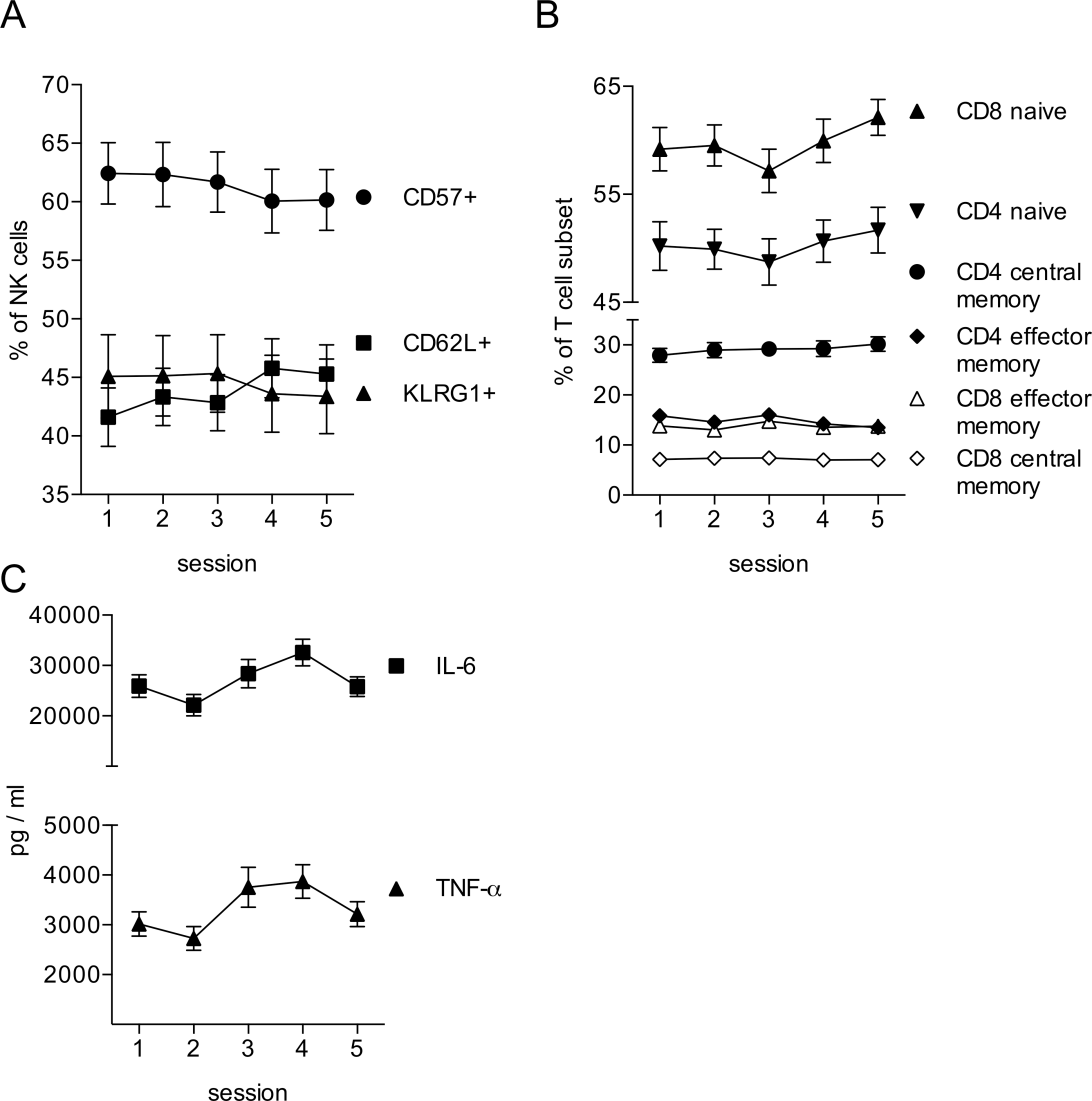
Lymphocyte subsets and monocyte reactivity during the exam period. The relative size of lymphocyte subpopulations was analyzed by multicolor flow cytometry. All data are presented as means ± sem of 20 individual participants. Data were analyzed by repeated measures ANOVA. Data sets displaying significant differences between sessions are shown in black. (A) NK cells (CD56+CD3- PBMC) were analyzed for the expression of CD57, KLRG1 and CD62L. (B) T cells (CD56-CD3+ PBMC) were pre-gated based on the expression of CD4 or CD8. Subpopulations were identified as CD45RA+CD62L+ (naive), CD45RA-CD62L+ (central memory) or CD45RA-CD62L- (effector memory). (C) Whole blood samples were treated for 3 h with LPS and samples were assayed for the production of IL-6 and TNF-α by monocytes. Data are presented as means ± sem of 20 individual participants. Data were analyzed by repeated measures ANOVA.

The immunophenotyping via flow cytometry allowed us to perform a more detailed analysis of the NK cells. This analysis revealed that in addition to the reduction in NK cell numbers, there was also a shift among the different NK cell subpopulations. We observed a significant reduction in the percentages of CD57^+^ (F(4, 76) = 5.52 p = .001; post-hoc LSD tests demonstrated a significant decrease from Session 1 to Sessions 4 (p = .005) and 5 (p = .004), a significant decrease from Session 2 to Sessions 4 (p = .001) and 5 (p = .004), and from Session 3 to Sessions 4 (p = .020) and 5 (p = .045)) and KLRG1^+^ NK cells (F(4, 76) = 4.11 p = .005; post-hoc LSD tests revealed a significant decrease from Session 1 to Session 5 (p = .029), a significant decrease from Session 2 to Sessions 4 (p = .029) and 5 (p = .022), and from Session 3 to Sessions 4 (p = .016) and 5 (p = .001)) when comparing the sessions before with the sessions after the exam period (Fig 3A). Conversely, we found an increase in CD62L^+^ NK cells over the observation period (F(4, 76) = 5.93 p = .000; post-hoc LSD tests revealed a significant increase from Session 1 to Sessions 4 (p = .002) and 5 (p = .001), a significant increase from Session 2 to Session 4 (p = .019), and from Session 3 to Sessions 4 (p = .007) and 5 (p = .006)). CD57 and KLRG1 are markers found on more mature NK cell subpopulations, whereas CD62L marks the more immature NK cells [42]. This indicates that the exam period induced a shift from mature to more immature NK cells in the blood samples. Interestingly, we observed a similar shift towards more immature cells within the T-lymphocyte compartment [43]. Over the observation period we detected an increase in the percentages of naïve CD4 T helper cells (F(4, 76) = 2.97 p = .025; post-hoc LSD tests showed a significant decrease from Session 3 to Sessions 4 (p = .031) and 5 (p = .002)) and cytotoxic CD8 T cells (F(4, 76) = 2.63 p = 0.41; post-hoc LSD tests showed a significant decrease from Session 2 to Sessions 5 (p = .030)), while we observed a slight decrease in CD4 effector memory cells (F(4, 76) = 5.72 p = .000; post-hoc LSD tests demonstrated a significant decrease from Session 1 to Sessions 4 (p = .018) and 5 (p = .002), and a significant decrease from Session 3 to Sessions 4 (p = .012) and 5 (p = .000)) (Fig 3B).

Stimulation of blood samples with lipopolysaccharide (LPS) induces the release of pro-inflammatory cytokines by monocytes [44]. In our analysis this response increased during the observation period, reaching the highest release of IL-6 (F(2.545, 48.346) = 7.6 p = .001; post-hoc LSD tests revealed a significant decrease from Session 1 to Session 2 (p = .029), a significant increase from Session 1 to Session 4 (p = .001), a significant increase from Session 2 to Sessions 3 (p = .023), 4 (p = .000) and 5 (p = .040), and a decrease from Session 4 to Session 5 (p = .002)) and TNF-α (F(4, 76) = 5.9 p = .000; post-hoc LSD tests revealed a significant increase from Session 1 to Session 3 (p = .034), a significant increase from Session 2 to Sessions 3 (p = .013), 4 (p = .000) and 5 (p = .031), and a decrease from Session 4 to Session 5 (p = .039)) at session 4 directly at the end of the exam period (Fig 3C). This is particularly interesting, as we observed a reduction of monocyte numbers during the observation period (Fig 2). Therefore, the enhanced cytokine release is not due to an increase in cell numbers, but it must be due to an enhanced reactivity of the monocytes. At session 5, one week after the exam period, both the monocyte numbers and the LPS-induced cytokine release showed a drop (Fig 3C).

**Fig 4:**
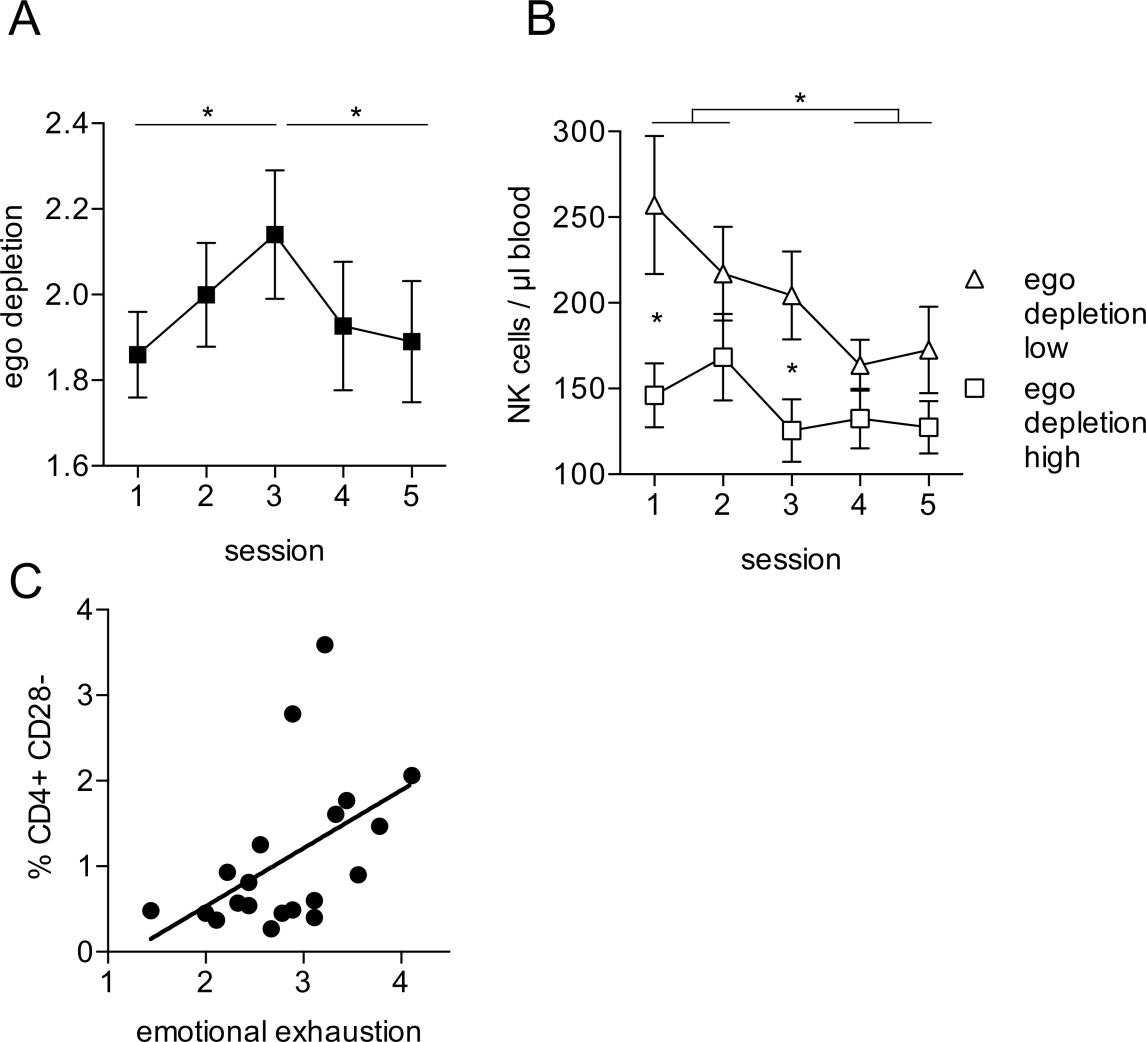
Immune parameters are associated with psychological variables. (A) Mean score for ego depletion measured at session 1–5. (B) Participants were divided at the median of ego depletion scale into high and low ego depletion groups. Data are presented as mean ± sem of 20 individual participants. (C) T cells were identified as CD56-CD3+ PBMC. The percentage of CD4+CD28- cells was associated with the reported level of emotional exhaustion at session 1. Data are presented as mean of 20 individual participants. Data were analyzed by correlation analyses.

We also analyzed the repeatedly measured psychometric scales in the participants for which a complete set of immune parameters was available. Only ego depletion showed a significant change over the observation period (F (4, 72) = 4.062 p = .005). The mean score significantly increased from Session 1 to Session 3 (p = .010), and showed a decrease from Session 3 to Session 5 (p = .007), such that self-reported ego depletion achieved its maximum during the examination week and decreased after examinations (Fig 4A). Although non-significant, we observed a similar trend for negative affect. For emotional coping, active coping, positive affect, and expectation of success, we observed a tendency towards a decrease from session 1 to session 3 with and an increase from session 3 to session 5. However, these changes were not significant.

Next we were interested to analyze possible relationships between the immunological and psychometric variables. When looking at the drop of NK cell numbers in the blood samples during the observation period, we noticed an interesting trend. NK cell numbers seemed to be different between participants reporting high ego depletion compared to individuals with low ego depletion. The drop in NK cell numbers during the observation period was only evident in individuals with low ego depletion, as participants with high ego depletion seemed to have already lower NK cell numbers at session 1 (Fig 4B). Although the difference failed to reach significance in our comparatively small sample size (F(4, 72) = 2.2 p = 0.078) post-hoc tests revealed significant mean differences between the NK cell counts in low and high ego depletion group at session 1(p = .022) and also at session 3 (p = .022), indicating that an interaction trend we found for NK cell counts and ego depletion is not solely due to the baseline differences at session 1. More important, session-wise mean comparisons within each group showed a significant decline of NK cell counts from Sessions 1 and 2 to Sessions 4 and 5 (1 to 4 p = .028; 1 to 5 p = .03; 2 to 4 p = .047) for the low ego depletion group, and no significant changes in the high ego depletion group.

An increase in CD28 negative T cells has been associated with chronic inflammatory conditions [45]. Interestingly, we observed a significant correlation between the percentage of CD28 negative CD4 T cells and emotional exhaustion (r = .50 p = .025) (Fig 4C).

Our psychometric measures determined different facets of chronic and acute stress as well as coping with stress. This inevitably yielded a number of measures that were in part highly intercorrelated. Therefore, we aggregated our psychological variables by means of a Factor Analysis (FA). We submitted participants' mean scores on the scales Emotional Exhaustion, Depersonalization, Depressive Symptoms, Self-control Capacity, Expectation of Success, Active Coping, Ego Depletion, Negative Affect, Positive Affect, and Emotional Coping to a principal components analysis (PCA) with orthogonal VARIMAX rotation. This yielded a two-factor solution accounting for 63% of the variance. Table 1 summarizes the loadings of all variables on the two uncorrelated factors. According to the observed pattern, factor 1 lends itself to an interpretation in terms of chronic stress. Thus, variables being commonly associated with chronic stress like Emotional Exhaustion, Depersonalization, Depressive Symptoms, Ego Depletion, and Negative Affect load positively on this factor with weights > .46, while Positive Affect and Emotional Coping load with high negative weight on this factor. This first factor accounted for 31.9% of the variance.

**Table 1:** Rotated component matrix.

Variables	Component	
	1	2
Emotional Exhaustion	.742	-.489
Depersonalization	.865	.059
Depressive Symptoms	.809	-.419
Self-control Capacity	-.304	.554
Expectation of Success	-.182	.847
Active Coping	-.085	.704
Ego Depletion	.468	-.733
Negative Affect	.575	-.591
Positive Affect	-.549	.326
Emotional Coping	-.508	.423

Psychological variables were factor analyzed using principal component analysis with Varimax (orthogonal) rotation. The analysis yielded two factors explaining a total of 63% of the variance for the entire set of variables. Factor 1 was conceptualized as ‘chronic stress’ due to positive loadings (>.46) by the variables Emotional Exhaustion, Depersonalization, Depressive Symptoms, Ego Depletion, and Negative Affect. This factor accounted for 31.9% of the variance. Factor 2 was labeled ‘effective coping with stress’ due to positive loadings (>.32) of the scales Self-control Capacity, Expectation of Success, Active Coping, Emotional Coping, and Positive Affect. The variance explained by this factor was 31.1%.

Factor 2, which is characterized by large positive loadings of the scales Self-control Capacity, Expectation of Success, and Coping (all > .5), as well as moderately positive loadings of Positive Affect and Emotional Coping and moderately negative loadings of Emotional Exhaustion and Depressive Symptoms, suggests an interpretation in terms of the potential to effectively cope with stress. This factor accounted for 31.1% of the variance.

**Fig 5:**
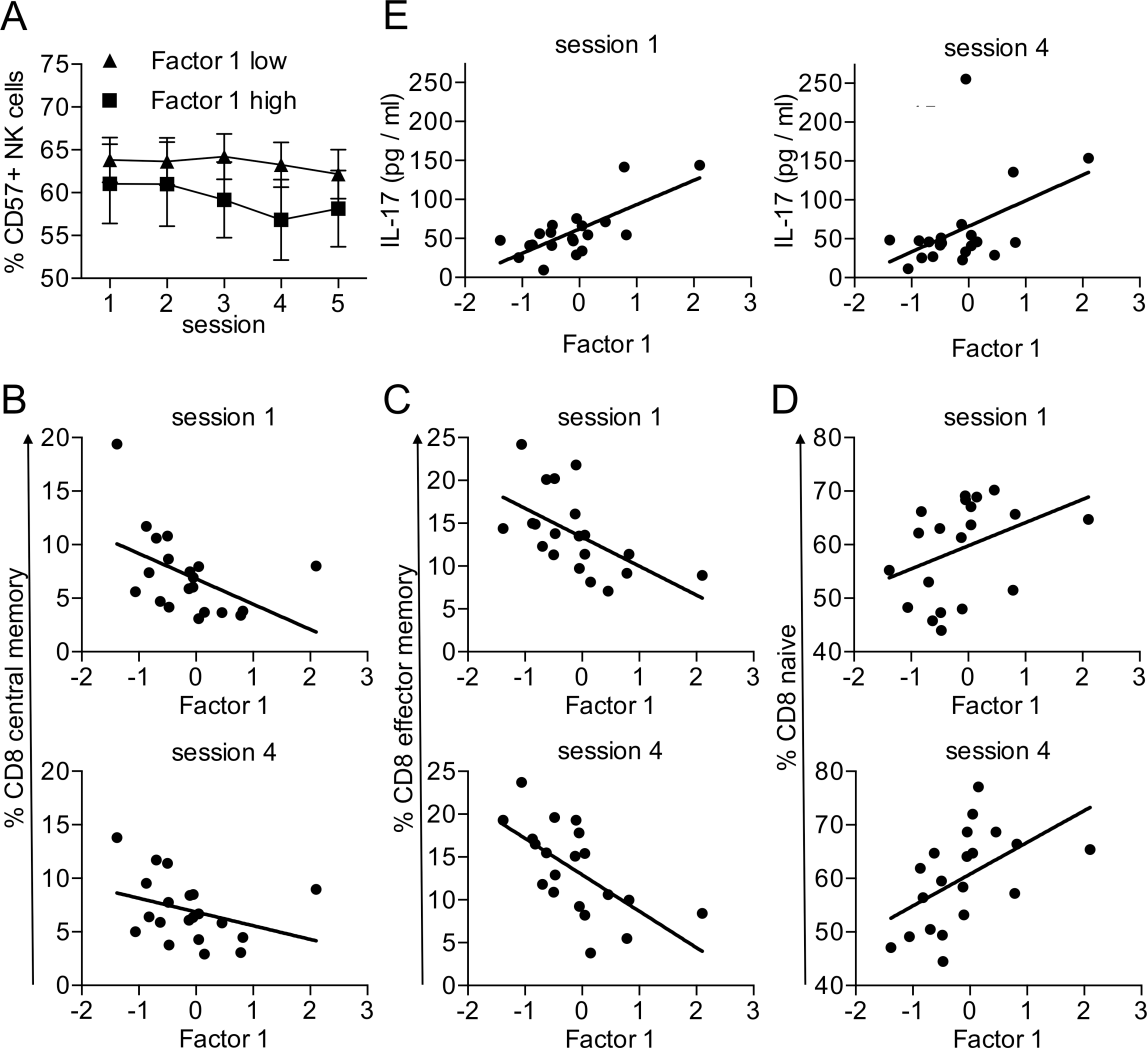
Association of maturation markers and inflammatory cytokines with chronic stress. (A) Participants were divided at the median score of Factor 1 into high and low Factor 1 groups. Data are presented as mean ± sem of 20 individual participants. Data were analyzed by 2x5 ANOVA with Factor 1 as between-participants factor and the five-level within-participants factor representing Session. CD8+ T cell subpopulations were identified as described in Fig 3. Percentage of central memory (B), effector memory (C) and naive (D) CD8 T cells at sessions 1 and 4 was associated with Factor 1. Data were analyzed by correlation analyses. Data from 20 participants are shown. (E) Serum concentration of IL-17 at sessions 1 and 4 was associated with Factor 1. Data were analyzed by correlation analyses.

We did not detect any systematic relationships between factor 1 psychometric score and changes over time of salivary cortisol measures. Changes over time in isolated psychometric scores also did not show any systematic relationships to cortisol levels. Next we investigated relationships between the immunological parameters and the two factors of the aggregated psychological variables. Factor 2 (‘effective coping with stress’) was not significantly correlated with immunological parameters. In contrast, we found that factor 1 (‘chronic stress’) had an impact on the shift towards more immature lymphocytes during the observation period. The reduction of the more mature CD57^+^ NK cells was more pronounced in participants with a high score on factor 1 (Fig 5A), suggesting that the acute stress of the exam period had more impact on individuals with higher chronic stress (F(4, 72) = 2.8 p = .04). We observed no significant mean differences between low and high chronic stress group for %CD57 NK cells at any session. Session-wise mean comparisons within each group showed a significant decline of % CD57 NK cells from Session 3 to Session 5 (p = .011) in the low factor 1 group, and a significant decline of % CD57 NK cells from Session 1 to Session 4 (p = .001), from Session 1 to Session 5 (p = .020), from Session, from Session 2 to Session 4 (p = .000), and from Session 2 to Session 5 (p = .043) in the high chronic stress group. Similarly, we observed a correlation between factor 1 and the proportion of naïve versus memory CD8 T cells. Already at session 1 the percentage of central (r = -.48 p = .034) and effector memory CD8 T cells (r = -.56 p = .010) was negatively correlated to factor 1 (Fig 5B and 5C). Therefore, high chronic stress seems to be correlated with a reduction in memory CD8 T cells. Conversely, we observed a trend towards a positive correlation between factor 1 and naïve CD8 T cells at session 1 (r = .38 p = .103) (Fig 5D). This correlation between the ratio of memory versus naïve CD8 T cells and factor 1 was still detectable at session 4 directly after the exam period (r_CD8central memory_ = -.34 p = .146; r_CD8 effector memory_ = -.64 p = .003; r_CD8 naive_ = .52 p = .019) (Fig 5B–5D).

Chronic stress has been associated with increased levels of pro-inflammatory cytokines [46]. In our analysis we found a positive correlation between the factor 1 and an increase in serum IL-17 levels (Fig 5E). Factor 1 and serum IL-17 levels correlated both at session 1 (r = .73 p = .000) and at session 4 (r = .45 p = .047), suggesting that chronic stress had a stronger impact on IL-17 levels then the acute stress of the exam period.

## Discussion

In the current study we used academic stress as a naturalistic stressor to investigate the effects of psychological stress on a wide range of immune parameters and assessed psychological variables associated with chronic stress and coping. Our objective was to investigate how examination stress will affect the immune response and whether these effects would be influenced by psychological variables.

Results indicated that absolute salivary cortisol levels measured after awakening and 30 minutes after awakening increased during examinations compared to the non-examination period. Although the elevation was significant only for cortisol levels after awakening, the pattern we observed was at least partially in line with existing data from examination stress protocol studies [39, 40, 47]. Indeed, the estimated effect size for absolute cortisol levels elevations was medium and also in accord with previous research [39, 40, 47]. Given the small and homogeneous sample size, and possible confounders (e.g. season, sleep duration), we cannot reliably judge the severity of stress added during examination stress. Contrary to our expectation, we observed a significant decrease of CAR. This is most likely due to the fact that an elevation of cortisol levels was more pronounced for the time after awakening and not for the time 30 minutes after awakening, and CAR was calculated as a difference between the cortisol levels after awakening and cortisol levels 30 minutes after awakening.

The decrease in the absolute numbers of NK cells is consistent with previous studies [1] and has been associated with chronic stress [48, 49]. However, the shift from mature to more immature NK cell subpopulations in peripheral blood has not been reported so far. This suggests that the brief naturalistic stressor results in a redistribution of specific lymphocyte subsets. Mature NK cells are likely being recruited into the tissue or adhere to the endothelium, which would explain the reduction in NK cell numbers and the shift towards an increased proportion of immature NK cells. We recently showed that the activation of the integrin LFA-1 is enhanced in mature NK cells, which would support their preferential adhesion and redistribution from the blood into tissues [50]. This lymphocyte redistribution could be interpreted as a functional response to recruit effector cells to locations where they may be needed in case of injury and infection.

Interestingly, we observed a similar redistribution towards more naïve cells in the T cell compartment. This suggests that a mobilization of mature and memory lymphocytes may be a general response towards stress. This is also supported by our finding that factor 1, as a parameter for chronic stress, was also correlated with a shift towards more naïve and less memory CD8 T cells.

The reduction in monocyte numbers could similarly be a result of the redistribution of these cells from the blood into the tissue. However, we also detected an increased functionality of these cells. Therefore, despite lower monocyte numbers we detected more LPS-induced production of IL-6 and TNF-α in response to the examination stress. Therefore, a brief naturalistic stressor may boost the functionality of monocytes. Monocyte responses are important for immune reactions against bacterial infections and NK cells and cytotoxic T cells are necessary for the defense against viral infections. Therefore, the redistribution of these cells towards possible sites of injury and infection and the enhanced function of monocytes may be a way to boost the immune system in response to the brief stressor and to protect the individual from infections. Interestingly, anticipatory stress as in the case of a preparation for an important exam has been associated with an immune-mediated protection from infection [51, 52]. Individuals are often able to work with high performance even if they are already in poor health, and then fall ill when the important exam is over [53]. We observed a drop in monocyte numbers and functionality in the last sample, one week after the exam period, which may indicate that the boost in immune function is reduced at this time point.

We assumed that there might also be individual differences beyond the examination period as a result of personality characteristics related stress and coping that may influence the immune parameters. Therefore, we tested for such relationships and obtained numerous correlations between immunological parameters and psychological variables. Factor 1, which represents variables commonly associated with chronic stress, positively correlated with serum levels of interleukin IL-17, which is secreted by Th17 cells. Th17 cells are important for immune responses against infections, particularly at mucosal surfaces. However, they are also associated with autoimmune diseases and chronic inflammatory disorders [54–56]. Th17 cells have not been investigated in chronic stress models so far. As psychological stress is associated with chronic inflammation, Th17 might be one of the biological links between chronic stress and inflammation. Previous studies reported that brief naturalistic stress can cause a shift from Th1 cytokines towards Th2 cytokines [2]. We did not observe such a shift in our data. On explanation for this discrepancy may be that other studies [12] measured cytokine concentrations after ex vivo stimulation of blood samples, while we measured cytokine levels in serum without stimulation.

Our data illustrate that psychological variables assessed via self-report (i.a. earlier experienced stress) influence the magnitude of immune reactivity induced by brief naturalistic stress. High level of chronic stress (as subsumed under factor 1) was associated with the reduction in the percentage of mature CD57^+^ NK cells, whereas no such association could be observed in the group with low level of chronic stress. This result is partially consistent with the study of Brosschot et al. that showed a greater reduction of the percentage of mature CD57^+^ NK cells during the stress situation in individuals with greater life stress [57]. Although the results of Brosschot et al. refer to immune reactivity to acute laboratory stressor, and chronic stress was conceptualized in terms of daily hassles in the last 2 months, there was some consistency in the results indicating that accumulated stress could alter the vulnerability of the individual to psychological stressors, which normally would not change the immune status.

In contrast, the drop in absolute NK cell numbers was more pronounced in individuals with low ego depletion. In individuals experiencing high levels of ego depletion the absolute NK numbers were already at a lower level and did not decrease much further. Interestingly, ego depletion was the only psychological variables intended to capture psychological states, which exhibited a clear-cut dependence on the time course of examination stress. In experimental and social psychology ego depletion refers to a state of impaired self-control. In particular, ego depletion effects were first demonstrated in experimental procedures using self-control tasks. The performance of participants who were required to engage in a self-control task earlier in the experiment was poorer relative to a control group which was not engaged in a self-control task in the first phase but performed a control task not related to self control instead [58, 59]. Furthermore, research has also shown that self-reported ego depletion was associated with increased perceptions of fatigue, effort, negative mood, and reduced glucose blood level [see [60] for review].

From an ecological immunology perspective, organismic energy availability impacts immune function due to energetic demands of immunity [61]. As animal models show, caloric restriction and reductions in body fat led to suppression of immune functions and increased risk of infections [62]. Interpreting our finding in this line would mean that high ego depletion results in lower energy levels in the body. As a consequence, NK cell-immunity may be suppressed in order to preserve energetic costs. Inadequate stress response to examination stress as a result of earlier experienced stress in highly ego depleted individuals could be another explanation of our results [63]. To our knowledge, there have been no studies addressing the relationship between ego depletion and NK cell numbers.

In addition, our data show that these psychological characteristics might be relevant not only in a stress context but also for predicting basal immune status [64]. Therefore, considering the impact of personality characteristics in the future can contribute to better understanding of personality driven immunity, and it can help to identify the responsible mechanisms.

There were no systematic associations between cortisol levels and subjective chronic stress experience as captured by Factor 1. We neither detected systematic significant correlations between percentage change values from ego depletion and percentage change values from three cortisol measures (wakening, wakening +30 and CAR) at sessions 1 and 3, and 5 and 3, such as elevations in cortisol seem to be independent from of psychological measures. We cannot completely delineate the causes for these results, because they can be due to other biochemical mechanisms underlying psychological stress-related measures. Another possible explanation is that questionnaires on stress-related psychological measures are not a good measure for subjective stress experience [39, 47]. The studies on the extent to which psychological stress measures are predictive for cortisol reactivity have also demonstrated inconsistent results [41].

### Limitations

Our study has several limitations. First, we were able to obtain complete data sets from only a relatively small sample of participants. This restriction may be responsible for null findings (reduced power). Likewise, we examined a rather large number of variables, which inevitably entails a risk of false positives. Our use of factor analysis reduced this problem at least on the psychological side. Nevertheless, one should be aware that, in line with the exploratory nature of these analyses, our findings should be considered as tentative and are in need of replication. Second, reflecting the gender distribution among psychology students in Germany, 85% of our participants were female which is problematic for the generalizability of our results. Gender may moderate the effects of stress on immunity by virtue of the effects of sex hormones on immunity [2]. Studies demonstrated that men are considered to be more biologically vulnerable [65]. Furthermore, although we controlled for health status, additional environmental factors, such as seasonal influences or reduced sleep duration, could have interfered with the results. However, despite these limitations, we believe that our study makes a significant contribution to understanding the effects of naturalistic stress on several immune functions including NK and T cell subsets.

## Supporting information

S1 Fig Cortisol response during study period. Cortisol concentration in saliva was measured in the evening as control, after awakening, and 30 min after awakening. Further, Cortisol Awakening Response (CAR) was calculated. Data are presented as mean ± sem of 29 individual participants for which a complete set of cortisol concentration data was available. Data were analyzed by repeated measures ANOVA.
(TIF)
