# Supplementary figures and images for: Impact of chronic and acute academic stress on lymphocyte subsets and monocyte function
Source: PLoS One. 2017 Nov 16;12(11):e0188108. doi: 10.1371/journal.pone.0188108 (PMC5690587; doi:10.1371/journal.pone.0188108)

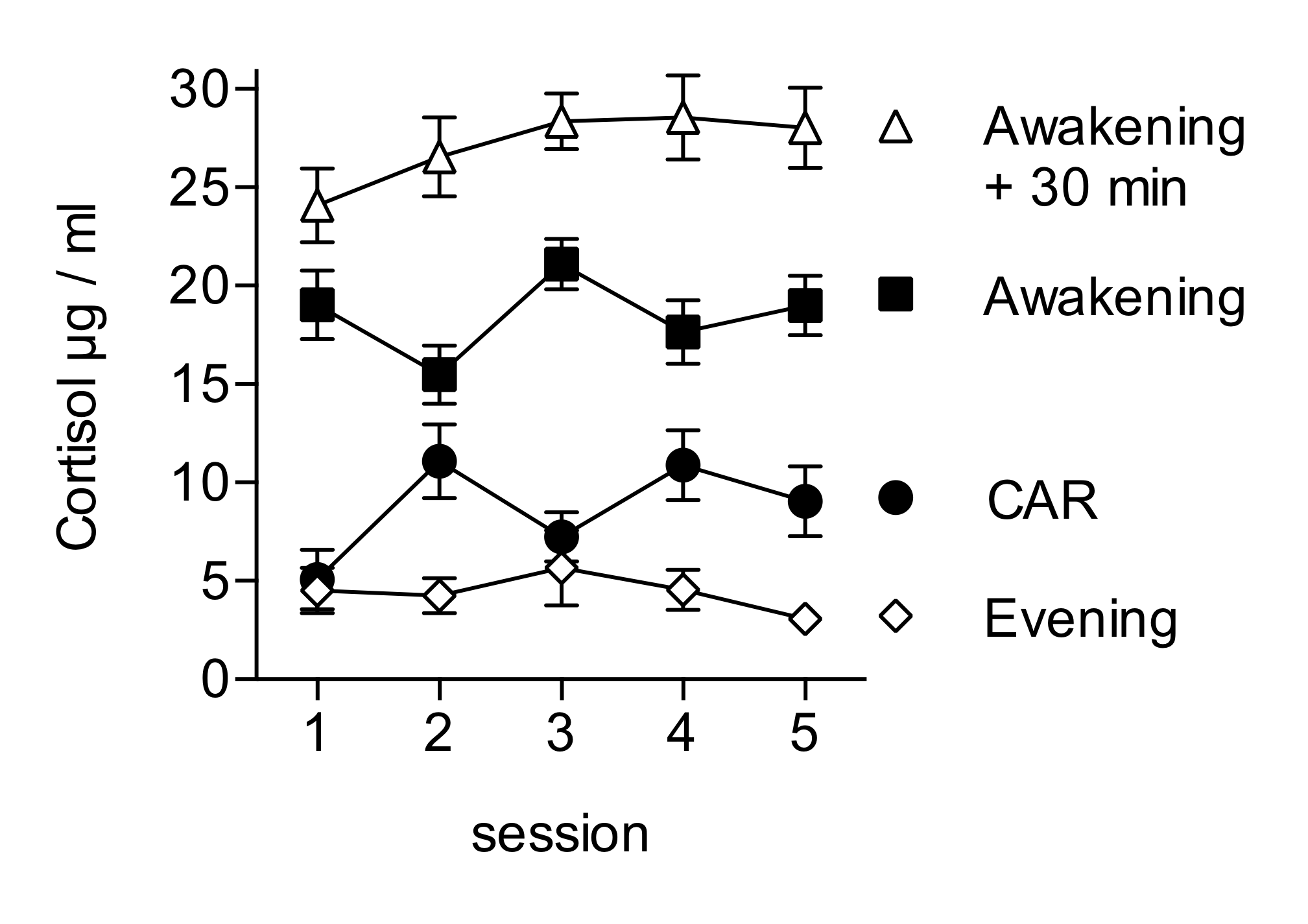

Supplement: S1 Fig — Cortisol concentration in saliva was measured in the evening as control, after awakening, and 30 min after awakening. Further, Cortisol Awakening Response (CAR) was calculated. Data are presented as mean ± sem of 29 individual participants for which a complete set of cortisol concentration data was available. Data were analyzed by repeated measures ANOVA. (TIF) [file pone.0188108.s001.tif]
